# Urban Care Farming to Enhance Quality of Life Among Older Adults: Protocol for a Waitlist Randomized Trial

**DOI:** 10.2196/78584

**Published:** 2026-02-25

**Authors:** Cynthia Chen, Jocelin Lam, Zhenye Shi, Katika Akksilp, Su Aw, Mary Foong-Fong Chong, Choon Nam Ong, Angelia Sia, Leng Leng Thang, Xin Kai Tham, JunXiang Pong, Kartini Omar, Joan Hung, Marek Kukumberg, Roger Ho, Elizabeth Diehl

**Affiliations:** 1 Saw Swee Hock School of Public Health, National University of Singapore and National University Health System, 12 Science Drive 2, 09-01T, Singapore, 117549, Singapore, (65) 6601 5526; 2 Schaeffer Center for Health Policy and Economics, University of Southern California, Los Angeles, CA, United States; 3 Health Intervention and Technology Assessment Program, Bangkok, Thailand; 4 Centre for Urban Greenery and Ecology Research, National Parks Board, Singapore, Singapore, Singapore; 5 Faculty of Arts & Social Sciences, National University of Singapore, Singapore, Singapore; 6 Terrapy SG, Singapore, Singapore; 7 Hort Therapeutics, Singapore, Singapore; 8 Healthy Longevity Translational Research Programme, Yong Loo Lin School of Medicine, National University of Singapore, Singapore, Singapore; 9 Division of Life Science (LIFS), Hong Kong University of Science and Technology (HKUST), Clear Water Bay, Hong Kong, China (Hong Kong); 10 Department of Environmental Horticulture, University of Florida, Gainesville, FL, United States

**Keywords:** biopsychosocial health, complex intervention, healthy and active aging, quality of life, urban care farming

## Abstract

**Background:**

Population aging poses challenges to health systems and costs, and evidence shows that older adults spend a long time in ill health. Improving healthspan, time spent in good health, allows older adults to contribute and improve in their quality of life. Active and healthy aging are crucial to improving healthspan. Urban care farming (UCF) is a behavioral intervention that is purported to enhance active and healthy aging.

**Objective:**

This trial evaluates the effectiveness of a care farming intervention in improving the quality of life and biopsychosocial health outcomes of older participants.

**Methods:**

We conducted a parallel group, 2-arm pragmatic waitlist randomized trial with a 1:1 allocation, in which participants were randomized into either the intervention or waitlist control arm. Community-dwelling participants aged 50-85 years, without any mobility issues, were recruited. Participants in the intervention arm commenced the 24-week UCF program, while waitlist control participants received no intervention during this period. The primary (World Health Organization Quality of Life−brief version) and secondary outcomes were collected at baseline, 6th month, and 12th month after the intervention group completed the trial. Secondary outcomes include objectively measured physiological outcomes, cognition, frailty, and self-reported psychosocial outcomes. Intervention effects were estimated using mixed-effects difference-in-differences regression to account for repeated measurements.

**Results:**

The randomized controlled trial commenced in April 2024, with the intervention group starting first. By April 2024, we had enrolled 137 participants at commencement, with 67 participants randomized to the intervention group and 70 to the control group. The intervention arm started in April 2024 and concluded in September 2024. Baseline data were collected in March 2024, and 6-month follow-up data were collected in September 2024. The waitlist control participants began the UCF intervention at the end of September 2024 and concluded in April 2025. Data collection for the 12-month follow-up concluded in May 2025. Analysis of the baseline and 6-month follow-up data is still ongoing.

**Conclusions:**

The outcomes of this study will contribute to the understanding of UCF on quality of life and health. This trial has potential positive implications for public health, as it utilizes a robust research design and methods to provide empirical insights into the multifaceted health benefits of the multicomponent UCF intervention. This trial could also serve as a model for future intervention research on scalable, community-based programs. Taken together, the UCF content and the outcomes, process, and economic evaluations completed through this study could inform scalable models of the UCF intervention, with potential implications for public health strategies to address health issues related to population aging.

## Introduction

Globally, population aging has raised concerns about the growing burden on health care systems and rising health care costs. While life expectancy has increased, individuals are living longer in poorer health [1]. Aging brings about physiological changes, such as the loss of muscle mass, which leads to functional decline and reduced brain volume [2,3], contributing to cognitive impairment [4]. Emotional and psychosocial well-being are also impacted, with older adults experiencing higher rates of depression [5] and social isolation [6]. In Singapore, 1 in 5 older adults aged 75 years and above shows signs of depression [7], and the proportion who committed suicide rose from 23% in 2000 to 30% in 2014 [8]. Furthermore, many mental health conditions remain underdiagnosed and untreated, largely due to stigma and delayed health-seeking behavior [9]. Active and healthy aging are crucial to improving healthspan. Care farming is a behavioral intervention that is purported to enhance active and healthy aging.

Care farming is a nature-based therapeutic intervention that integrates agricultural and farming practices to positively influence participants in multiple health domains [10-12]. Nature-based intervention (NBI) has garnered traction as a green social prescribing activity that addresses upstream social determinants of health [13]. The nature-based biopsychosocial resilience theory (NBRT) provides a conceptual foundation for understanding how exposure to nature can promote health and well-being [14]. The NBRT posits that humans have an inherent affinity for the natural environment, through which they derive restorative benefits that support biopsychosocial resilience and overall health. According to White and colleagues, biopsychosocial health restoration occurs through increased exposure to nature, which reduces stress (eg, by mitigating pollution through the use of trees) while simultaneously strengthening resources such as physical fitness, social connections, and self-esteem. The NBRT also aligns with the World Health Organization’s quality of life (QOL) definition, which emphasizes health and illness as outcomes of complex interactions among biological, psychological, and social factors.

The World Health Organization defines QOL as an individual’s perception of their position in life, considered within the context of their culture, values, goals, expectations, standards, and concerns [15]. To capture this multidimensional construct of health, the WHOQOL (World Health Organization Quality of Life) instruments assess multiple domains, including physical, psychological, social, and environmental aspects of well-being. From this perspective, a comprehensive understanding of a person’s health or QOL requires the consideration of biological processes and lifestyle; cognitive and emotional functioning; coping capacities; and the social context of relationships, support systems, and culture. Given the multidimensional nature of WHOQOL, its domains align closely with the components of the NBRT, offering a coherent framework for evaluating health and well-being. In line with this, active aging aims to maintain functional ability, promote participation, and enhance the QOL as people age [16,17].

Growing evidence suggests that adopting healthy behaviors can lower disease risk, enhance physical and mental health function, and delay the onset of care dependency [18]. Care farming is valued for its multidimensional impact on biopsychosocial health and QOL. It is an intervention with the goal of promoting holistic health through activities by increasing light-to-moderate physical activity; reducing stress, anxiety, and negative mood; enhancing positive mood; fostering social interaction and sense of purpose; stimulating cognition; and encouraging healthier eating habits [19-24]. Unlike many NBIs, which can be undertaken individually, care farming places a stronger emphasis on fostering community engagement and collaboration. Hence, we hypothesize that care farming better addresses psychological and social relationship domains of QOL by fostering a sense of purpose and social connectedness through collective cultivation, responsibility, and group harvesting and communal cooking. Care farming is therefore a highly complex intervention consisting of interrelated components that target multiple domains of well-being. This is consistent with the UK Medical Research Council’s framework for complex interventions, which considers the interaction of multiple components, variability in outcomes, and context-sensitive implementation [25].

Although care farming is garnering attention as a green care intervention, the empirical evidence remains limited. Systematic reviews by Murray et al [12] and Coventry et al [26] highlight limitations in current literature, concluding that there is mixed evidence showing that care farming or NBIs improved individuals’ health, well-being, and QOL. Murray et al also found that only 2 care farming studies employed randomized controlled methods, with the majority of reviewed studies being pre-post and qualitative studies conducted with small sample sizes. Moreover, care farming tasks are typically tailored to the needs and capabilities of participants, shaped by the type of farm and the resources available. Such flexibility poses challenges for research, as the heterogeneity of care farming interventions complicates the identification of mechanisms of change and limits both replicability and generalizability. Both reviews also noted that care farming is grouped with other NBIs, making it difficult to attribute observed outcomes specifically to farming-related activities. These limitations underscore a clear literature gap and the need for a robust, well-designed experimental study with structured tasks to establish the effectiveness of care farming as a health intervention.

There is a strong theoretical and empirical rationale to assess its potential impacts across physical, psychological, cognitive, and behavioral domains. This study aims to (1) develop a structured care farming intervention, which we termed urban care farming (UCF) and (2) evaluate the acceptability and effectiveness of a structured UCF program for older adults in Singapore using a waitlist randomized controlled trial. Specifically, this outcome evaluation seeks to answer the following research questions:

1. Effectiveness on primary outcome: Does participation in UCF improve overall QOL, as measured by the WHOQOL-BREF (World Health Organization Quality of Life−brief version), relative to a waitlist control?
2. Effects on secondary and exploratory outcomes: What are the effects of UCF on physical activity, psychosocial well-being, dietary behaviors, cognitive function, and biomarkers?

These research questions were guided by the intervention’s logic model (Figure S1 in Multimedia Appendix 1), which outlines hypothesized pathways from program activities to short- and medium-term outcomes. This intervention targets multiple dimensions of health, including physical, psychological, social, and cognitive outcomes, aligning with both the WHOQOL framework and biopsychosocial model [27,28]. In addition, subsequent, separate evaluations of UCF will also incorporate process and cost-effectiveness assessments to further examine implementation effectiveness and identify challenges. These evaluations include the following:

1. Process evaluation will be conducted to assess the quality of urban care farming (UCF) intervention delivery, including fidelity, dose delivered and received, acceptability, and to identify the mechanisms and contextual factors that facilitate or hinder outcomes.
2. Cost-utility analysis will be conducted from the provider and societal perspective to assess the potential value of scaling up the UCF intervention, using quality-adjusted life years (QALYs) as the primary effectiveness metric. This economic evaluation will be exploratory in nature and designed to inform future trial design and policy translation.

## Methods

### Study Design

This UCF intervention was a parallel group, 2-arm, pragmatic waitlist randomized trial with 1:1 allocation ratio to either intervention or waitlist control. The intervention group completed the first 24-week UCF program, followed by a 24-week observation period. The control arm served as a waitlist group, receiving no intervention during the initial 24 weeks but undergoing the same intervention in the following 24 weeks after the intervention group completed. Participants in both arms completed assessment at baseline, 6-month, and 12-month follow-up. We reported using the standard protocol items: recommendation for interventional trials (Standard Protocol Items: Recommendations for Interventional Trials [SPIRIT]) checklist (Checklist 1) [29].

### UCF Intervention

The UCF intervention was designed as a structured 24-week care farming curriculum and delivered by certified horticulturists and trained farmers. It was intended to engage older adults with the natural environment through therapeutic farming (eg, nature walks around sensory gardens and reflecting on nature landscape) and gardening activities (eg, plant cultivation and gardening) in green spaces, with the goal of enhancing QOL across physical, psychological, and social domains of health. Both the intervention and waitlist control arms participated in a weekly UCF program, lasting between 2.5 and 3 hours per session over 24 weeks. The program included activities such as fertilization, sowing, watering, transplanting, maintaining, and harvesting vegetables and herbs. The UCF intervention included structured, facilitated sessions involving a range of agricultural and food-growing activities, such as seedling propagation, composting, harvesting of edible greens, and hands-on food preparation using farm-grown produce. These went beyond casual gardening and were intentionally designed to promote physical activity, encourage teamwork and autonomy, and stimulate cognitive and sensory engagement. Each participant selected 1 weekday to attend weekly. All sessions offered each week were identical in content. For example, group 1 attended a Monday session for 24 weeks. This arrangement was necessary as the intervention site could not accommodate all 60-70 participants, depending on the study arm. Furthermore, smaller group sizes promoted meaningful discussion and the forming of social networks. Treatment adherence was monitored using attendance sheets. Additionally, treatment fidelity was used to monitor and ensure the consistency of intervention content delivery.

Trainers facilitated the 24-week UCF intervention, engaging them with horticultural and urban care farming activities to acquire relevant skills and knowledge. To ensure consistent content and activities delivery, trainers were provided training materials, which listed the objectives and activities (Supplementary 1 in Multimedia Appendix 1). Participants received guidance on fundamental planting techniques, information on different varieties of plants, and their nutritional value. UCF-integrated activities related to gardening and farming that fostered social connections and promoted psycho-emotional well-being were also included in the intervention. Additionally, participants gained insights into the benefits of naturally grown food without chemical additives and were encouraged to make healthier dietary choices. This intervention included other activities such as cooking, nutrition, and arts and crafts. Table 1 presents the overview of the activities that participants engaged in the intervention.

A small incentive of S$10 (US$7.35) [30] per session was reimbursed to participants to cover travel costs to the intervention site. Participants were given vegetable packs fortnightly in addition to crops they had personally cultivated. This hands-on experience provided farm-to-table experience. The practice also encouraged participants to reflect on the quality of their produce.

### Study Setting

The study was held in the western region of Singapore, at a national garden equipped with growing edibles and therapeutic gardens, and a rooftop community garden. Enrolled participants began the intervention at the national garden, which includes foundational knowledge and hands-on training on plants and farming. As part of the intervention, they transitioned to a community rooftop garden, collaborating to grow, maintain, and harvest plants.

The UCF intervention was designed as a structured 24-week care farming curriculum and delivered by certified horticulturists and trained farmers. It was intended to engage older adults with the natural environment through therapeutic farming (eg, nature walks around sensory gardens and reflecting on nature landscape) and gardening activities (eg, plant cultivation and gardening) in green spaces, with the goal of enhancing QOL across physical, psychological, and social domains of health. Both the intervention and waitlist control arms participated in a weekly UCF program, lasting between 2.5 and 3 hours per session over 24 weeks. The program included activities such as fertilization, sowing, watering, transplanting, maintaining, and harvesting vegetables and herbs. The UCF intervention included structured, facilitated sessions involving a range of agricultural and food-growing activities, such as seedling propagation, composting, harvesting of edible greens, and hands-on food preparation using farm-grown produce. These went beyond casual gardening and were intentionally designed to promote physical activity, encourage teamwork and autonomy, and stimulate cognitive and sensory engagement. Each participant selected 1 weekday to attend weekly. All sessions offered each week were identical in content. For example, group 1 attended a Monday session for 24 weeks. This arrangement was necessary as the intervention site could not accommodate all 60-70 participants, depending on the study arm. Furthermore, smaller group sizes promoted meaningful discussion and the forming of social networks. Treatment adherence was monitored using attendance sheets. Additionally, treatment fidelity was used to monitor and ensure the consistency of intervention content delivery.

### Inclusion and Exclusion Criteria

Participants who enrolled in the study met these inclusion criteria: (1) aged 50 and 85 years, (2) willing to take part in either the intervention or waitlist control arm, (3) agree for their data to be used for research purposes, and (4) ambulant to be able to carry out gardening tasks (eg, watering the plants). Participants were excluded if they had medical conditions that could compromise or interfere with their ability to participate, upper or lower disabilities that limit their mobility, or were unwilling to provide their medical or health records for research.

### Ethical Considerations

The study has been approved by the National University of Singapore Institutional Review Board (NUS-IRB-2023‐191). The approval has also been granted for the pilot study of this project. Any modification to the approved protocol will be submitted for review by the ethics committee. If there are any amendments, changes will be reflected in the trial registry. All participants provided written consent prior to the participation. Data collected was anonymized and kept confidential. Consent from participants was obtained to publish the results from deidentified data. To acknowledge the time and effort required, participants were provided with a S$30 (US $22.05) [30] honorarium for each data collection session (approximately 210 min). The amount was set at a level appropriate to compensate participants without constituting undue inducement, consistent with ethical guidelines for research involving human participants. Participants’ data (eg, case record forms, laboratory tests, information sheets, and consents) are stored in a locked cabinet in a researcher’s office. All other data (eg, biomarker results, and responses to surveys) have been deidentified. All personal identifier data (eg, names and contact information) are kept separate from the research data by a trusted third party. All data collection has been completed, and a link (otherwise known as code key) was passed to the trusted third party so the research team only works with deidentified data. After the study has been completed, all personal data will be kept for a maximum of 5 years. Research and biological data will be kept for a maximum of 10 years. During the study, the data will only be accessible to the research team. The research team has exclusive rights to the deidentified data for 36 months after the trial is completed.

### Dissemination

In addition to disseminating our research findings to the funder of this study, we will disseminate our findings to other countries, the study participants, and the research community. We will also follow the authorship guidelines of the International Committee of Medical Journal Editing (ICMJE) [31].

**Table 1.** Summary of indoor and outdoor care farming activities across the 24-week intervention.

No. of sessions	Program description	Activity breakdown	Goals of intervention
15 sessions	Participants engaged in indoor plant- and farming or gardening-related knowledge-sharing sessions led by trained horticulturist farmers. Participants learned basic skills and knowledge in maintaining a garden and acquired skills in pruning, soil mixing, transplanting, and composting, as well as knowledge of the properties of plants and vegetables. Example topics included composting, the properties and nutritional value of plants and vegetables, and gardening techniques. A few indoor sessions also included microgreen planting, which participants brought home to tend to, as well as nature-art activities designed to foster creative expression and social interaction. Following the indoor activities, participants attended outdoor hands-on sessions where they engaged in green maintenance in the designated garden or farm area and practiced the gardening techniques introduced during the indoor sessions. Several outdoor sessions included therapeutic walks, during which participants took part in guided educational strolls through therapeutic gardens to learn about the unique properties of local plants and to strengthen their connection with nature. During these walks, participants engaged in sensory-based activities involving touch, scent, and visual observation, which created opportunities to reconnect with local flora and fauna and to reminisce. Several of the 15 sessions at the national garden focused on nutrition, cooking, and consumption of herbs, vegetables, and fruits. Participants learned about the nutritional value and importance of consuming more plant-based diets. Live cooking demonstrations were provided, and participants sampled the cooked vegetables. Participants were encouraged to share their recipes to promote greater social interaction.	Indoor and outdoor activities: 20 minutes of briefing of daily activities 60 minutes of indoor gardening and sharing of farming tips 60 minutes of outdoor gardening activities with short breaks in between 10 minutes of debriefing Nutrition and cooking activities: 20 minutes of briefing of daily activities 50 minutes of gardening, harvesting, and preparation 60 minutes of cooking and demonstration, food sharing, and social interaction 20 minutes of debriefing	Increase physical activity Improve gross and fine motor skills Strengthen working memory Gain a sense of achievement Care for plants—promote sense of purpose, meaningfulness, and hope Improve nutrition knowledge and attitude toward vegetable consumption Improve interpersonal interaction
8 sessions	These 8 sessions were held at the community garden near the national garden. Care farming activities involved participants working collaboratively to plan, carry out, and care for crops from initial germination through to harvest in the community garden. Participants also prepared meals together using the produce they planted and harvested.	15 minutes of briefing of daily activities 120 minutes of gardening and horticultural activities with short breaks in between 15 minutes of debriefing	Increase physical activity Improve physical performance Strengthen working memory Gain a sense of achievement Care for plants—promote sense of purpose and meaningfulness Improve gross and fine motor skills Improve community engagement and social interaction
1 session	Participants organized a collective indoor community event to showcase their harvest and promote community gardening activities. Participants from different session days came together to attend this event. Trainers assisted participants in preparing for the community event.	150 minutes of preparation, sharing, and exchange among participants and urban farming experts and horticulturists	Improve place attachment Improve community engagement and social interaction

### Outcome Measures

Outcome domains were selected based on the intervention’s theoretical framework and logic model (Figure S1 in Multimedia Appendix 1) and included primary outcomes of QOL, along with secondary and measures capturing psychosocial, behavioral, cognitive, and biological responses.

### Primary Outcome

The primary outcome of this study was participants’ QOL using the WHOQOL-BREF (World Health Organization Quality of Life−brief version), a multidimensional QOL tool developed by the World Health Organization. The instrument consists of 25 questions evaluating 4 domains of QOL, namely physical (7 items), psychological (6 items), social relationships (2 items), and environment (8 items). One item in the social dimension—“How satisfied are you with your sex life?”—was removed because it was considered culturally sensitive for this study population. The items that were negatively worded had their scores inversed. In addition, there were also 2 questions about general QOL and health satisfaction questions. The physical health domain measures individuals’ biological well-being related to the body and its functions. The psychological domain examines the aspects of mental well-being and cognitive functioning. Social relationships were based on interpersonal connections and support systems. The environment domain covers factors related to surroundings and external conditions [32]. Higher scores in each of the 4 domains indicate better QOL. The score of the 4 domains was summed together, where a higher score indicated a higher QOL. The validity and reliability of the WHOQOL-BREF were tested in Singapore in Chinese, English, and Malay. The instrument is valid and reliable for assessing the QOL among English-, Chinese-, and Malay-speaking people in Singapore [33].

### Secondary Outcomes

#### Physiological Outcomes

The physiological component included changes in cardiometabolic outcomes. The cardiometabolic outcomes consisted of the following physical measurements: (1) waist circumference and hip circumference, (2) systolic and diastolic blood pressure, (3) resting heart rate, (4) body weight, and (5) height and BMI. Weight and height were measured without shoes. BMI was calculated by dividing weight in kilograms by the square of height in meters. Systolic and diastolic blood pressures were measured after the participants had rested for at least 5 minutes [34]. Waist circumference (cm) was measured at the middle point between the lowest rib in the standing position and the iliac crest.

#### Frailty

Participants’ frailty was assessed using the Fried frailty phenotype [35], which consists of 5 components: unintentional weight loss, exhaustion, weakness, slowness, and low physical activity. Each of the components was calculated using the computation methods described in [35, 36]. Weight loss was a self-reported item to determine if participants had lost 4.5 kg or more in the past year. Exhaustion was assessed through 2 self-reported feelings of fatigue and low energy. Physical activity was evaluated based on self-reported items on walking for 30 minutes or more a day and the amount of time spent sitting. Weakness was measured using grip strength, with scores computed according to gender and BMI-specific cut-offs, where values below the threshold indicated weakness. Slowness was assessed using a 5-m walking speed test, with thresholds for slowness determined according to gender and height; scores above the threshold indicated slowness. Frailty scores were computed and categorized into non-frail (none or 1 criterion met), pre-frail (2 criteria met), and frail (3 or more criteria met) [35, 36].

#### Biomarkers

Fasting blood was collected and sent to a certified laboratory. Around 10 mL of blood was collected in each data collection. In addition, clinical bioprofiling, such as the assessment of plasma glucose, hemoglobin A_1c_, and lipid panels (total cholesterol, triglyceride, low-density lipoprotein cholesterol, and high-density lipoprotein cholesterol), and serum creatinine were examined. Plasma biomarkers were analyzed using commercially available enzyme-linked immunosorbent assay and bead-based multiplexing Luminex system immunoassay kits. Evaluated analytes involved several functions: inflammation, immune system adaptation, senescence, and cellular signaling. More specifically, the targeted biomarkers assessed cytokines involved in innate immune functions (interleukin-1 receptor antagonist, interleukin-1 beta, interleukin-6, interferon-gamma-induced protein 10, tumor necrosis factor alpha), adaptive immunity (interferon gamma, interleukin-10, interleukin-13, interleukin-17A), macrophage inflammatory markers involved in aging altering cellular communication and inflammatory state (macrophage inflammatory protein-3 beta, stromal cell-derived factor-1 alpha, matrix metalloproteinase-3, matrix metalloproteinase-9, monocyte chemoattractant protein-1, plasminogen activator inhibitor-1, granulocyte-macrophage colony-stimulating factor, vascular endothelial growth factor A), and chemokines (eotaxin, interleukin-8). Study participants with abnormal biomarkers were encouraged to consult with a physician.

#### Psychosocial Outcomes

A range of psychosocial measures was adopted. To measure social well-being, the Lubben social network scale [37] was used. The De Jong Gierveld Loneliness Scale [38], which has 6 items, was used to measure social and emotional loneliness. The Brief Sense of Community 8-item scale was employed to assess participants’ perceptions of utilizing community resources to fulfill their needs [39]. Older adults’ resilience was measured using the Brief Resilience Scale [40]. The 10-item Perceived Stress Scale was adopted to measure psychological stress [41].

#### Cognitive Outcomes

The Montreal Cognitive Assessment (MoCA) was adopted to evaluate cognitive function, with assessment categorized into memory, executive functioning, attention, language, visuospatial skills, and orientation domains [42]. The maximum total score was 30, with a cutoff of 26 or above indicating a normal cognitive function. Additionally, functional near-infrared spectroscopy (fNIRS) was collected. fNIRS measures hemodynamic changes in the cerebral cortex over time, reflecting neuronal activity via neurovascular coupling. Near-infrared light penetrates tissues to the cortex, where it is absorbed by oxyhemoglobin and deoxy-hemoglobin. Increased neuronal activity will lead to a net rise in oxy-hemoglobin and a slight decrease in deoxy-hemoglobin, making oxy-hemoglobin a marker of cortical activity. Participants were asked to perform a semantic verbal fluency task during each fNIRS measurement. The verbal fluency task consisted of alternating blocks of 4 control periods and 3 task periods. Each control and task period was 30 seconds long. Participants were instructed to say the names of the weekdays consecutively and generate words that belong to a particular category (eg, fruits, flowers, professions) during task periods [43]. Categories differed between task periods and fNIRS measurements. The total number of words generated per category was noted.

#### Dietary Outcomes

Participants’ attitude and behavior toward healthier diet consumption, for example, consumption of fruits and vegetables, were measured. Participants’ healthy eating behavior was also assessed by indicating the frequency of consuming food from various groups (eg, vegetables, fruits, fried snacks, sweet snacks, fast food, sweet drinks, fruit juice) and their willingness to consume fruits and vegetables. Attitude toward healthy eating was measured by an 11-item scale [44].

#### General Life Satisfaction

A single-item measure, “In general, how satisfied are you with your life as a whole?”, was adopted. Participants ranked a 10-point scale, with 1 being “very unsatisfied” and 10 being “very satisfied.” The single-item satisfaction with life measure is a validated instrument that performs similarly to the multiple-item satisfaction with life scale [45].

### Exploratory Outcomes

Physical activity was measured with Fitbits. The intervention group was given a Fitbit Inspire 2 or Inspire 3 device to track their step count throughout the trial. Fitbit is an activity device that uses a tri-axis accelerometer to provide real-time feedback and allows self-monitoring of steps. Participants were encouraged to wear the device as frequently as possible to obtain the most accurate results. Fitbit intraday data were downloaded, providing minute-by-minute data that was available through a web application programming interface.

### Sample Size

This study aimed to compare the QOL between the treatment and waitlist control participants, using a 1:1 allocation ratio. Based on a prior study of the overall WHOQOL measure, if the true difference-in-differences estimate between experimental and control means is 4.93 with a standard deviation of 9.29 [46], a sample of 57 intervention and 57 control subjects is required to reject the null hypothesis based on a 2-tailed significance level of 5% and power of 80%. We inflated the sample by 20% to account for dropouts and the potential loss of participants due to noncompliance and faulty measurements. Accounting for all these factors, the total sample size required for 2 arms is 138 participants.

### Recruitment

Singapore residents were invited to participate in this study. We adopted a 2-prong recruitment strategy. First, we collaborated with local community partners and senior activity centers (SACs) to recruit potential participants. The findings from our feasibility study suggest that older adults are more inclined to participate when approached through familiar and trusted sources. Concurrently, we simultaneously conducted open recruitment by promoting the study via social media and word of mouth. Although this second approach engaged young-old adults, some have encouraged their parents to join the study. Our recruitment approaches ensured effective outreach to our target audience of older adults. Recruitment occurred between February and March 2024. During the recruitment phase, the research team was in close contact with SACs and community partners to facilitate recruitment. During recruitment, interested participants, recruited from either partnered SACs or online recruitment, provided their contact details on a sign-up form. Subsequently, a research staff member contacted these participants to share more information about the study and screened them first for their age eligibility. Those who met the criteria were invited to a briefing session where more detailed information about the research was presented. Informed consent was obtained from participants who agreed to participate and were enrolled in this research study. Participation was voluntary, and participants could withdraw from the study at any time without reason or penalty.

### Blinding and Assignment of Groups

Participants were randomized into either of the groups based on a computer-generated random generator using Stata 14 [47]. A first block of 130 participant IDs was randomly generated, followed by a second block of 20 due to additional recruitment. At baseline data collection, all participants and data collectors were blinded to group allocation. Randomization and allocation results were shared only at the last station after written informed consent and the completion of all baseline data to ensure allocation concealment. To maintain neutrality, the research staff who generated the randomization were independent of the staff responsible for revealing group allocations to participants. As the nature of the intervention required participants to be involved in the study, both participants and research staff were eventually aware of group allocation. All outcome assessments were conducted by trained assessors who were independent of intervention delivery. Assessors were blinded to participant group allocation at baseline and 6-month and 12-month follow-up, where feasible. However, full blinding could not always be maintained during follow-up, particularly when participants disclosed their participation during data collection. To mitigate potential bias, the assessors were trained to ensure accurate and objective data collection, and validated instruments were used to reduce subjectivity in cognitive and psychosocial outcomes.

### Data Collection and Analysis

#### Data Collection

Baseline data were collected after informed consent was obtained. Table 2 outlines the measures and instruments that were collected in each of the 3 assessment time points. At baseline (T0) and 6-month follow-up (T1), participants completed the survey, blood test, fNIRS, MoCA, and physiological assessments at the National University of Singapore. At the 12-month follow-up (T2), all the assessments completed at T0 and T1 were repeated, except for the blood test and fNIRS. Participants were invited to return to the same location for T2 data collection. However, we anticipated that some participants may be unwilling or unable to return in person. For those who did not attend the in-person assessment, a survey link was sent to them to allow them to self-administer remotely. The rest of the assessment was not collected from these participants. To maximize the data collected, participants were reimbursed a small amount of S$30 (US $22.05) [30] each for T0 and T1 assessments, and S$20 (US $14.70) [30] for T2 in-person assessment or S$10 (US $7.35) [30] for completing the T2 online self-administered survey. The physiological assessments were collected by trained research assistants, while blood samples were collected by certified phlebotomists or registered nurses. The survey was available in English and Chinese and was self-administered by participants; however, trained research assistants assisted participants who were illiterate in either of the languages to complete the survey. fNIRS data were collected by a trained engineer.

**Table 2.** Overview of study outcomes and measurement instruments used.

	Feasibility	Baseline	6-month	12-month
Demographics				
Age	✓	✓	✓	
Gender	✓	✓	✓	
Ethnicity	✓	✓	✓	
Marital status	✓	✓	✓	
Education	✓	✓	✓	
Pre-existing diseases, self-reported		✓	✓	✓
Smoking and alcohol consumption	✓	✓	✓	
Community participation	✓	✓	✓	
Quality of life: WHOQOL-BREF^a^		✓	✓	✓
EQ-5D-5L	✓	✓	✓	✓
Psychosocial				
Lubben social network scale	✓	✓	✓	✓
De Jong Gierveld loneliness scale	✓	✓	✓	✓
Brief sense of community		✓	✓	✓
Brief resilience scale	✓	✓	✓	✓
Perceived stress scale	✓	✓	✓	✓
Satisfaction with life	✓	✓	✓	✓
Physical activity				
Fitbit tracking	✓	✓	✓	
Dietary				
Attitude toward consumption of fruits and vegetables	✓	✓	✓	✓
Fruits and vegetable consumption frequency (behavior)	✓	✓	✓	✓
Cognition				
Functional near-infrared spectroscopy		✓	✓	
Montreal cognitive assessment	✓	✓	✓	✓
Physiological outcomes				
Body weight and height	✓	✓	✓	✓
Waist and hip circumference	✓	✓	✓	✓
Blood pressure	✓	✓	✓	✓
Resting heart rate	✓	✓	✓	✓
Fried phenotype of frailty		✓	✓	✓
Biomarkers				
Fasting plasma glucose, HbA_1c_^b^		✓	✓	
Fasting insulin		✓	✓	
Lipid profile: triglyceride, total cholesterol, high-density lipoprotein cholesterol, low-density lipoprotein cholesterol		✓	✓	
Cytokines: interleukin-1 receptor antagonist, interleukin-1 beta, interleukin-6, interferon-gamma-induced protein 10, tumor necrosis factor alpha		✓	✓	
Chemokines: eotaxin, interleukin-8		✓	✓	
Adaptive immunity: interferon gamma, interleukin-10, interleukin-13, interleukin-17A		✓	✓	
Macrophage inflammatory markers: macrophage inflammatory protein-3 beta, stromal cell-derived factor-1 alpha, matrix metalloproteinase-3, matrix metalloproteinase-9, monocyte chemoattractant protein-1, plasminogen activator inhibitor-1, granulocyte-macrophage colony-stimulating factor, vascular endothelial growth factor A		✓	✓	

a WHOQOL-BREF: World Health Organization Quality of Life−brief version.

b HbA_1c_: hemoglobin A_1c_.

#### Data Management

All registered participants’ information was deidentified and given a unique identification number. This identification number was used on all data collected. All contact information of potential participants collected via in-person recruitment or online platforms was stored electronically and password protected. All survey data, whether completed in-person or remotely, were collected via a secure online link and stored electronically. Physiological data were initially recorded on paper and entered into an electronic database. fNIRS and MoCA assessments were conducted in person, and the resulting scores were entered and stored electronically. Blood samples were collected during in-person visits, deidentified, and immediately sent to accredited laboratories for analysis. The results were then stored electronically by the respective laboratories. All electronic records were stored on a secure institutional server, which was protected by password access and restricted to authorized personnel only. All personal information of potential or enrolled participants will be kept a maximum of 5 years before being discarded. The research data used in the publication will be kept for a maximum of 10 years before being discarded. If participants provide informed consent for future studies, their biological samples will be securely stored for a maximum of 10 years. Otherwise, all biological materials will be discarded immediately after the analyses for this study are completed.

#### Statistical Analysis

The primary analysis, intention-to-treat, will be conducted using a linear mixed effects model with overall WHOQOL score as the dependent variable and the interaction effect of intervention allocation and time as the independent variable. Domain-specific scores will also be analyzed. To address potential bias arising from missing data due to attrition or incomplete responses, our primary longitudinal analyses will employ linear mixed-effects models, which are robust to unbalanced data structures and can handle missing observations under the missing-at-random assumption. While listwise deletion may be used for cross-sectional comparisons or descriptive analyses, this will be clearly indicated. Secondary outcomes will be analyzed using similar methods. These analyses will be conducted at 6-month follow-up to investigate the effectiveness of the intervention as well as post-intervention behavioral maintenance at 12 months. Sensitivity analysis using the per-protocol approach will also be carried out. Baseline characteristics of participants who discontinued or were lost to follow-up will be compared to those who completed the study to investigate differential dropout between intervention and control groups.

Due to potential imbalances between the groups, further analysis techniques, such as adjustment or matching of baseline characteristics, will be used in the difference-in-difference analysis. Specifically, we will adjust or match on key baseline covariates (eg, age, gender, years of education, marital status, and housing type) to improve group comparability at baseline in case of any imbalance despite randomization. This approach improves covariate balance and enhances the comparability of groups prior to applying linear mixed models for outcome analyses. Conducting both intention-to-treat and per-protocol analyses for this behavioral intervention allowed comparison of the results and provided a comprehensive understanding of the treatment effect. All evaluations were and will be performed using a 2-sided test at the 5% level of significance.

#### Method Monitoring

There was no data monitoring board. The study team had complied with data collection and management procedures approved by the National University of Singapore ethics committee (NUS-IRB-2023‐191) and abided by the rules of medical confidentiality. We recognized the importance of safety monitoring in community-based interventions involving older adults. Accordingly, safety precautions and procedures were implemented in line with the university’s ethics committee. Trainers were briefed on these safety procedures, and, during sessions, they consistently reminded participants to exercise caution and check for potential tripping hazards while engaging in activities. Additionally, weekly post-intervention debriefings with trainers were conducted to identify any safety concerns or adverse events related to the outdoor farming sessions (Supplementary 2 in Multimedia Appendix 1). No adverse events were reported throughout the intervention, suggesting that it was conducted safely and with appropriate precautions in place. There was no independent audit of the trial.

To ensure consistency in intervention delivery, all trainers were provided with a structured facilitator manual that outlined session-by-session objectives, activities, and key content to be delivered. Teaching presentation slides were also shared with all trainers. Additionally, training sessions were conducted prior to the start of the intervention to align all trainers on core delivery principles, safety protocols, and communication style. To ensure fidelity, staff completed checklists after each session to document adherence to the protocol and note any deviations. Additionally, a member of the research team attended a random sample of sessions for verification purposes. Weekly debriefing meetings were conducted among the research team and trainers to determine whether the intervention was executed as planned, create feedback loops, and allow the early identification of problems and problem-solving to enhance implementation. We have included this fidelity assessment document in Supplementary 3 in Multimedia Appendix 1.

### Economic Evaluation

A cost-utility analysis will be conducted from the provider and societal perspective to assess the potential value of scaling up the UCF intervention, using QALYs as the primary effectiveness metric. This economic evaluation will be exploratory in nature and designed to inform future trial design and policy translation.

Resource use associated with the UCF intervention will be systematically recorded, including staff time (trainers), materials (gardening supplies), venue rental, and transportation. Unit costs will be derived from internal budget records and prevailing trainers’ wage rates. Any health care utilization during the intervention period (eg, GP visits, hospitalizations) will be tracked using participant self-reports and costed using standard unit prices obtained from publicly available Ministry of Health fee benchmarks or institutional databases.

Effectiveness will be expressed in terms of QALYs, derived from utility scores measured using the EQ-5D-5L instrument at baseline and postintervention. Area under the curve methods will be applied to calculate QALYs over the 24-week period. The decision tree model will be developed using the TreeAge software to compare the two scenarios: (1) the waitlist control group and (2) the urban care farming program. An incremental cost-effectiveness ratio (ICER) will be calculated as the ratio of the change in cost over QALY. The outputs of these analyses will be costs, QALY, and the incremental cost-effectiveness ratio to reflect the program’s cost-effectiveness. As Singapore does not have an official willingness-to-pay threshold for QALYs, a cost-effectiveness acceptability curve will also be plotted to show the probability that the intervention is cost-effective at varying willingness-to-pay thresholds.

## Results

**Figure 1.**
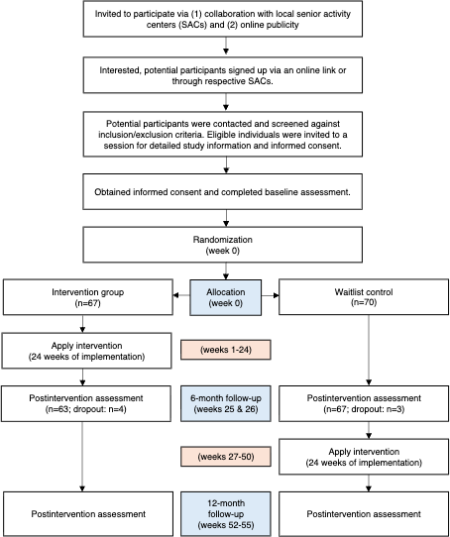
Illustration of the participant flow including the time schedule of enrollment, intervention, and assessments. Sample sizes (n) reported above were based on intention-to-treat random allocation. A “dropout” was defined as a participant who voluntarily withdrew from the study or could not be recontacted, resulting in missing data.

This randomized controlled trial (RCT) commenced in April 2024, with the intervention group undergoing the intervention for the first 24 weeks, which ended in September 2024. Prior to the start of the data collection and intervention, participants were screened for eligibility, and informed consent was obtained. A total of 137 participants were enrolled, with 67 randomized to the intervention group and 70 to the control group. Baseline data were collected in March 2024, and the 6-month follow-up was completed in September 2024. The waitlist control group began the 24-week UCF intervention in late September 2024 and completed it in April 2025, with a 3-week break over Christmas, New Year, and Lunar New Year period. Data collection for the 12-month follow-up concluded in May 2025. Analysis of the baseline and 6-month follow-up data is currently ongoing. The participant timeline is presented as a flowchart in Figure 1.

## Discussion

This paper describes the design of a waitlist randomized trial to evaluate the effectiveness of UCF, a complex behavioral intervention, in improving the QOL among older adults. UCF leverages the principles of biophilia and therapeutic horticulture to promote active and healthy aging. Previous studies have suggested that nature-based interventions may provide benefits such as improved mood [48] and increased physical activity [21]. While pilot studies have been conducted to illustrate health benefits in Singapore [49,50], these studies are typically underpowered and not designed with scalability in mind, leaving gaps in understanding their long-term impact and cost-effectiveness. Moreover, the existing evidence remains mixed, highlighting the need for well-powered, methodologically robust trials to assess the effectiveness of care farming as a public health intervention.

Our study aims to address these gaps through a robust RCT design, incorporating a waitlist control group to strengthen causal inference. By including objective physiological outcomes (eg, BMI, blood pressure), validated psychological and cognitive measures, and QOL assessments aligned with the WHOQOL framework, the trial aims to generate comprehensive and policy-relevant evidence. This study is also timely and relevant to Singapore’s aging population and growing interest in a community-driven approach to aging in place. With growing public interest in urban farming, this intervention leverages that momentum to promote health through nature-based, skill-building activities that may be sustained beyond the program period. While our study has been designed for scale-up, this study primarily serves to evaluate acceptability and preliminary outcomes. Further research, including a planned cost-effectiveness and process evaluation, will be necessary to assess implementation feasibility and inform national policy translation.

Nevertheless, this study has several limitations. The effect size used for sample size estimation was based on a prior WHOQOL study, which may not fully reflect the characteristics of the Singaporean older population or the unique aspects of the UCF intervention. The calculated sample size also includes a 20% buffer to account for expected attrition in long-term community interventions. Sensitivity analyses for secondary outcomes were not included. One methodological challenge is estimating the effect sizes of repeated measures data. To address this, the study will employ linear mixed-effects models and robust standard errors to derive reliable difference-in-difference estimates. This statistical approach enables the evaluation of longitudinal data, accounting for within-subject correlation and heterogeneity in responses over time. Attrition over the 24-week period may limit the ability to evaluate longer-term outcomes, though mitigation strategies such as regular participant engagement and reminders have been implemented. Next, the use of self-reported measures may introduce recall and reporting bias; however, the inclusion of biomarkers and clinical assessments partially mitigates this. Finally, the findings may be less generalizable to more frail or homebound populations, as the study sample consists of relatively healthy, community-dwelling individuals who are able to travel and commit to a 24-week intervention. As such, the study sample is likely skewed toward relatively healthy and motivated individuals, which may limit the generalizability of our findings. While the results offer important insights into the potential benefits of urban care farming, future work should explore adaptations of the intervention to accommodate more vulnerable older adults and assess its effectiveness in those populations. For future trials, we also recommend a more structured approach to adverse event monitoring, including standardized incident reporting forms and real-time logs, to further strengthen safety assurance and protocol reproducibility.

In conclusion, this RCT evaluates the impact of a multicomponent UCF behavioral intervention on QOL, across physical, psychological, social, and environmental domains. We will also assess whether greater compliance with UCF sessions improves QOL, mental health, social outcomes, physical activity, and dietary attitudes toward consuming fruits and vegetables. If effective, this intervention could serve as a model for future community−based public health research targeting the QOL and well-being of older adults. The findings will also contribute to future health economic analyses and support evidence-based policy formulation under the national Age Well SG initiative.

## Supplementary material

10.2196/78584 Multimedia Appendix 1 Logic model (Figure S1), sample curriculum (Supplementary 1), and implementation monitoring sample documents (Supplementary 2 and 3) for urban care farming intervention (UCF).

10.2196/78584 Checklist 1 SPIRIT checklist.

## Abbreviations

fNIRS: functional near-infrared spectroscopy
MoCA: Montreal Cognitive Assessment
NBI: nature-based intervention
NBRT: nature-based biopsychosocial resilience theory
QALY: quality-adjusted life year
QOL: quality of life
RCT: randomized controlled trial
SAC: senior activity center
UCF: urban care farming
WHOQOL: World Health Organization Quality of Life
WHOQOL-BREF: World Health Organization Quality of Life−brief version
