# Supplementary material for: Urban Care Farming to Enhance Quality of Life Among Older Adults: Protocol for a Waitlist Randomized Trial
Source: JMIR Res Protoc. 2026 Feb 25;15:e78584. doi: 10.2196/78584 (PMC12935458; doi:10.2196/78584)
Supplement: Multimedia Appendix 1 [file resprot-v15-e78584-s001.docx]

**Supplementary Figure 1**. Urban Care Farming logic model


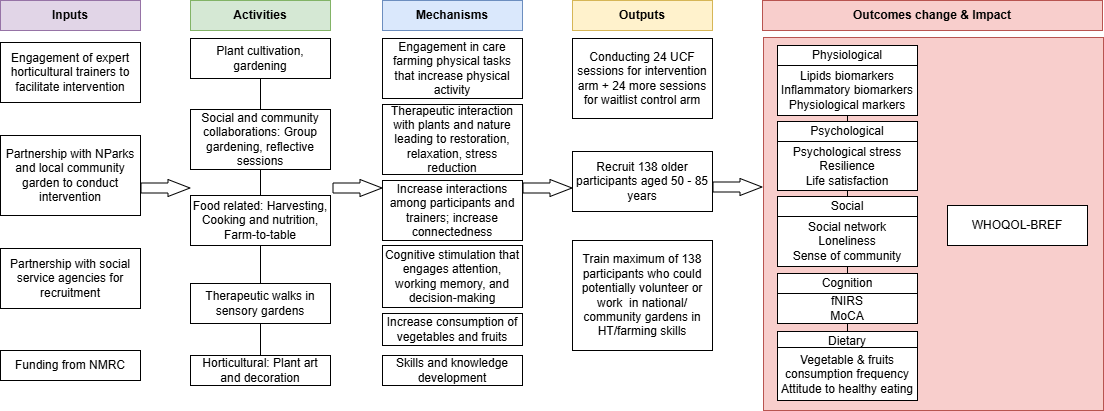


| **Supplementary 1**. UCF structured objective and planned curriculum for second week | |
| --- | --- |
| **Urban Care Farming Intervention Curriculum**  **WEEK 2** | |
| **Intervention objective** | 1. Examine the effects of UCF interventional activities on healthy longevity and improving older participants’ quality of life 2. Examine the impact of UCF on biological, psychological, and social changes across time and between groups 3. Examine the cost benefit and cost effectiveness of conducting UCF activities |
| **Specific learning objective** | 1. Identify and learn how to use 6 types of gardening tools 2. Identify 4 types of gardening protective clothing and its uses 3. Practice wearing the protective clothing introduced 4. Recognise that staying safe and healthy is part of the activity goals 5. Describe the benefits of maintaining tools & equipment 6. Highlight the importance of proper housekeeping |
| **Skills / Abilities acquired** | 1. Able to use and maintain basic gardening tools for their correct functions 2. Know the importance of wearing protective clothing for gardening activity 3. Basic understanding of safety hazards when involved outdoors |
| **Outcomes** | - Increased basic gardening knowledge - Increased physical activity - Strengthen working memory - Improve interpersonal interaction |

| **INSTRUCTIONS** |
| --- |

1. Follow the lesson plan if the weather is dry: **Part 1 > Part 2**
2. Wet weather contingency plan:
   - A short period or extended rain: **Part 1 > Part 2a**

| **LESSON PLAN** |
| --- |

|  | **Time** | **Instructions** |
| --- | --- | --- |
| **Pre-Class** | 15 min | Recap on last week’s topic  Brief on today’s timeline & learning objectives |
| **Part 1**  (Indoors) | 45 min | **Theory Lesson with Slides:**  Introduction to basic Gardening Tools and its Uses Maintaining Gardening Tools  Housekeeping Safety Precautions & Protective Clothing  *Participants should be given 5-10 min to rest and settle down before starting the lecture.* |
|  |  |  |
|  |  | By the end of this part of the session, participants would have:   1. Understood the safety practices 2. Been mentally prepared for practising using the tools |
| **Part 2**  (Outdoors) | 60 min | 1. **Warm Up Exercise** 2. **Practise wearing the protective clothing distributed**   *Ensure that everyone is geared up with sun hats and water bottles.* *Repeat the safety practices.*  - Hand Gloves - Arm Sleeves - Sun Hat - Covered shoes (by participant)   1. **Emphasise and repeat on the weather conditions and safety precautions** - Rest if tired - Drink more water - Call for help when needed 2. **Demonstrate the use of tools discussed** *Location: Tool Shed, near the trellis planters*  - Watering Cans - Watering Hose - Garden Hand Shovel - Garden Hand Fork - Changkol - Hand Secateurs   May allow them to take turns to practise the use of the changkol and hand secateurs on garden beds. Assess the group’s capacity from there.  Introduction to the rest of the alternative materials used including cotton gloves, plastic fork, mini bucket shovel and seedling lifter etc.   1. **Demonstrate maintaining of secateurs** Using sandpaper and WD-40 2. **Demonstrate proper housekeeping and waste management** - Washing & ~~Drying~~ drying of Tools - Sweeping - Location of throwing of inorganic wastes - Location of throwing of organic wastes - Location of throwing of food scraps   **May discuss any personal experience with tools that become spoiled or rusty after a long time.** A jumbo bag will be provided for horti-wastes instead of trash bags. |
| **Part 2a**  (Indoors - wet weather) | 45 min | Prepare a set of tools to pass around in class except the watering hose.   1. **Demonstrate the use of tools discussed**  - Watering Cans - Garden Hand Shovel - Garden Hand Fork - Changkol - Hand Secateurs - Sandpaper - WD-40 - Potted Plants  **Discuss any personal experience with tools that become spoiled or rusty after a long time.**  1. **Go through Wet Weather Slides:**   If there is more time:   - Demonstrate the Lux Meter Test indoors Purpose of Lux Meter: To measure light intensity. - Demonstrate the soil pH /moisture metre test indoors with a pot of plant Purpose of 3-in-1 metre: To measure moisture, pH and light.   *If the rain stops, proceed back to Part 2 to continue with outdoor activities.* |
| **After Class**  (post class) | 15 min | - Recap on today’s topic. - Answer relevant questions. - Brief on next week’s activity. - Remind them to bring their protective clothing every week. - Distribute vegetables to the participants |
| **Planning notes:** | | |
| - Allow ample time for participants to settle down between each part of the session. However, also keep track of the time so that there will be no overrun of sessions. This is to ensure all participants can benefit from the same contents and not leave before the sessions end if it overruns - **Assistant facilitator is to constantly document the session by taking photos of different activities in each session.* ***Also be reminded to take a group photo of all the research participants and both facilitators, as this will be used as documentation for service reimbursement*** - Passcode for Tool shed will be provided for the class - ***Review and record on the fidelity sheet after class** | | |
| **Interesting/useful facts:** | | |
| - Discuss about other tools that they have seen before and understand how they work too. - You can use baking soda and water or vinegar with salt to remove rust. Soak them for 12 hours. <https://www.youtube.com/watch?v=nFFiqwKCZvM> | | |

| **Summarised session and outcomes** | | |
| --- | --- | --- |
| **Activity** | **Objectives** | **Outcomes** |
| - Recap | - Allow participants to apply the skills learnt from last week | - Strengthen working memory |
| - Part 1 | - Identify and learn how to use 6 types of gardening tools - Identify 4 types of gardening protective clothing and its uses - Describe the benefits of maintaining tools & equipment | - Increased gardening knowledge |
| - Part 2 / 2a | - Practise using the protective clothing introduced - Practise how to use 6 types of gardening tools - Recognise that staying safe and healthy is part of the activity goals - Highlight the importance of proper housekeeping | - Increased basic gardening knowledge - Strengthen working memory - Increase physical activity - Interpersonal interaction - Improve interpersonal interaction |

| **SAFETY INSTRUCTIONS** |
| --- |

1. Take the concrete path to the edible demonstration garden.
2. If it is raining, walk slowly. Watch your step and be careful of tripping too.
3. If the weather is too hot, bring along your water bottles.
4. Raise your hands to call for help when not feeling well.
5. Before working physically, do some warm-up exercise.
6. Avoid long uncomfortable positions and repetitive moments - don’t squat for too long, don’t stand in the same position for too long, and don’t carry too heavy.
7. Use tools and gloves, not your hands.
8. Be careful not to injure yourself when using tools too.
9. Remember to wash your hands once you are done.
10. Rest if you are tired.
11. Always wear protective clothing when outdoors.

**Supplementary 2**. Discussion guides for weekly debriefing with trainers

**UCF Weekly Discussion/Updates**

**Program Progress and Updates**

- How did last week’s sessions go? Were there any noticeable successes or challenges?
- ⁠Did all planned activities go as expected? If not, what adjustments were needed?

**Participant Engagement and Response**

- How engaged were the participants during the sessions? Did they seem motivated and interested?
- ⁠Did any participants show enthusiasm or challenges with certain tasks?
- ⁠Did any participants need additional support, or was anyone showing signs of disengagement?

**Feedback on Curriculum and Activities**

- Do you have suggestions for adjusting the activities or curriculum to enhance engagement?
- ⁠Were specific materials or resources used to support learning outcomes?

**Operational and Logistical Challenges**

- Were there any issues with tools, materials, or the setup that affected the sessions?
- ⁠Did any environmental factors (like wet weather) impact the activities or safety? How were these managed?
- ⁠What can be done to improve logistics or preparation for upcoming sessions?

**Health and Well-being Observations**

- Have you noticed any positive changes in participants’ health or well-being (e.g., mood, physical activity)?
- ⁠Are there any participants who may benefit from additional resources or adjustments based on their physical or emotional needs?

**Open Feedback**

- Are there any specific stories or observations from this week that you think are important to share?
- ⁠Do you have any other suggestions for improving the program’s effectiveness or participant experience?

**Supplementary 3**. Treatment fidelity form

**Treatment fidelity for Urban Care Farming**

| **Name:** |  | **Role:** | **Trainer / Observer*** |
| --- | --- | --- | --- |
| **Date:** |  |  |  |
| **Weather:** | **Sunny / Rainy / Others*:** |  |  |

***Please delete where applicable**

Overall adherence to intervention content elements

1. **Evaluating overall adherence to intervention content elements:**
2. To what extent did the listed ***outdoor*** activities occur?

- Attempted, not able to complete, please state why:

__________________________________________________________________________

__________________________________________________________________________

- Completed
- Deviated, please state why:

__________________________________________________________________________

__________________________________________________________________________

- Skipped elements, which elements have been skipped:

__________________________________________________________________________

__________________________________________________________________________

**Reason/s for above choice: _____________________________________________________**

1. To what extent did the listed ***indoor*** activities occur?

- Attempted, not able to complete, please state why:

__________________________________________________________________________

__________________________________________________________________________

- Completed
- Deviated, please state why:

__________________________________________________________________________

__________________________________________________________________________

- Skipped elements, which elements have been skipped and why:

__________________________________________________________________________

__________________________________________________________________________

**Reason/s for above choice: _____________________________________________________**

1. **Duration of delivery**
2. Duration of ***outdoor*** activities

- Yes, the session was delivered as scheduled (e.g., scheduled 60 min and 60 min was completed)
- No, the session was delivered more than scheduled (e.g., scheduled 60 min and 75 min was completed)
- No, the session was delivered less than scheduled (e.g., scheduled 60 min and 45 min was completed)

Please state the reason for the increase/decrease in time spent on activities:

_________________________________________________________________________________

_________________________________________________________________________________

1. Duration of ***indoor*** activities

- Yes, the session was delivered as scheduled (e.g., scheduled 60 min and 60 min was completed)
- No, the session was delivered more than scheduled (e.g., scheduled 60 min and 75 min was completed)
- No, the session was delivered less than scheduled (e.g., scheduled 60 min and 45 min was completed)

Please state the reason for the increase/decrease in time spent on activities:

_________________________________________________________________________________

_________________________________________________________________________________

1. **Were participants given opportunities to work together?**

- Yes, to a large extent
- Yes, a small extent
- No

1. **Participants’ response**

- Overall participants responded very positively for this lesson
- Overall participants responded positively for this lesson
- Overall participants did not respond positively for this lesson

Additional remarks:

_________________________________________________________________________________

_________________________________________________________________________________

_________________________________________________________________________________
