# Supplementary material for: Urban Care Farming to Enhance Quality of Life Among Older Adults: Protocol for a Waitlist Randomized Trial
Source: JMIR Res Protoc. 2026 Feb 25;15:e78584. doi: 10.2196/78584 (PMC12935458; doi:10.2196/78584)
Supplement: Checklist 1 [file resprot-v15-e78584-s002.docx]

**
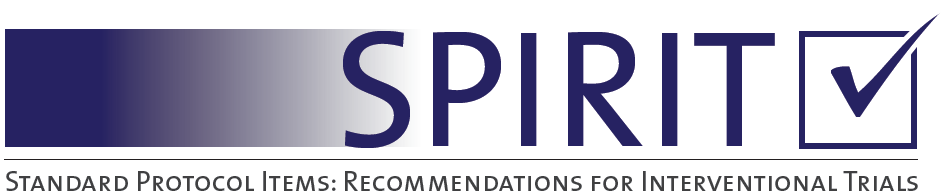
**Supplementary. SPIRIT checklist for UCF study

| Section/ item | | | Item No | Description | Addressed in sections | |
| --- | --- | --- | --- | --- | --- | --- |
| **Administrative information** | | | | |  | |
| Title | | | 1 | Descriptive title identifying the study design, population, interventions, and, if applicable, trial acronym | Title page | |
| Trial registration | | | 2a | Trial identifier and registry name. If not yet registered, name of intended registry | Abstract | |
|  |  |  | 2b | All items from the World Health Organisation Trial Registration Data Set | See table after SPIRIT checklist | |
| Protocol version | | | 3 | Date and version identifier | - | |
| Funding | | | 4 | Sources and types of financial, material, and other support | Funding statement | |
| Roles and responsibilities | | | 5a | Names, affiliations, and roles of protocol contributors | Title page | |
|  |  |  | 5b | Name and contact information for the trial sponsor | Funding statement | |
|  | | | 5c | Role of study sponsor and funders, if any, in study design; collection, management, analysis, and interpretation of data; writing of the report; and the decision to submit the report for publication, including whether they will have ultimate authority over any of these activities | Funding statement | |
|  | | | 5d | Composition, roles, and responsibilities of the coordinating centre, steering committee, endpoint adjudication committee, data management team, and other individuals or groups overseeing the trial, if applicable (see Item 21a for data monitoring committee) | - | |
| Introduction | | |  |  |  | |
| Background and rationale | | | 6a | Description of research question and justification for undertaking the trial, including summary of relevant studies (published and unpublished) examining benefits and harms for each intervention | Introduction | |
|  | | | 6b | Explanation for choice of comparators | Methods: Study design | |
| Objectives | | | 7 | Specific objectives or hypotheses | Methods: UCF Intervention | |
| Trial design | | | 8 | Description of trial design including type of trial (eg, parallel group, crossover, factorial, single group), allocation ratio, and framework (eg, superiority, equivalence, noninferiority, exploratory) | Methods: Study design | |
| Methods: Participants, interventions, and outcomes | | | | |  | |
| Study setting | | | 9 | Description of study settings (eg, community clinic, academic hospital) and list of countries where data will be collected. Reference to where list of study sites can be obtained | Methods: Study setting | |
| Eligibility criteria | | | 10 | Inclusion and exclusion criteria for participants. If applicable, eligibility criteria for study centres and individuals who will perform the interventions (eg, surgeons, psychotherapists) | Methods: Inclusion and exclusion criteria | |
| Interventions | | | 11a | Interventions for each group with sufficient detail to allow replication, including how and when they will be administered | Methods: UCF intervention | |
|  |  |  | 11b | Criteria for discontinuing or modifying allocated interventions for a given trial participant (eg, drug dose change in response to harms, participant request, or improving/worsening disease) | Methods: Recruitment | |
|  |  |  | 11c | Strategies to improve adherence to intervention protocols, and any procedures for monitoring adherence (eg, drug tablet return, laboratory tests) | Methods: UCF intervention | |
|  |  |  | 11d | Relevant concomitant care and interventions that are permitted or prohibited during the trial | - | |
| Outcomes | | | 12 | Primary, secondary, and other outcomes, including the specific measurement variable (eg, systolic blood pressure), analysis metric (eg, change from baseline, final value, time to event), method of aggregation (eg, median, proportion), and time point for each outcome. Explanation of the clinical relevance of chosen efficacy and harm outcomes is strongly recommended | Methods: Outcome measures | |
| Participant timeline | | | 13 | Time schedule of enrolment, interventions (including any run-ins and washouts), assessments, and visits for participants. A schematic diagram is highly recommended (see Figure) | Results:  Participant timeline  Fig. 1 | |
| Sample size | | | 14 | Estimated number of participants needed to achieve study objectives and how it was determined, including clinical and statistical assumptions supporting any sample size calculations | Methods: Sample size | |
| Recruitment | | | 15 | Strategies for achieving adequate participant enrolment to reach target sample size | Methods: Recruitment | |
| **Methods: Assignment of interventions (for controlled trials)** | | | | |  | |
| Allocation: | | |  |  |  | |
| Sequence generation | | | 16a | Method of generating the allocation sequence (eg, computer-generated random numbers), and list of any factors for stratification. To reduce predictability of a random sequence, details of any planned restriction (eg, blocking) should be provided in a separate document that is unavailable to those who enrol participants or assign interventions | Methods: Blinding and assignment of groups | |
| Allocation concealment mechanism | | | 16b | Mechanism of implementing the allocation sequence (eg, central telephone; sequentially numbered, opaque, sealed envelopes), describing any steps to conceal the sequence until interventions are assigned | Methods: Blinding and assignment of groups | |
| Implementation | | | 16c | Who will generate the allocation sequence, who will enrol participants, and who will assign participants to interventions | Methods: Blinding and assignment of groups | |
| Blinding (masking) | | | 17a | Who will be blinded after assignment to interventions (eg, trial participants, care providers, outcome assessors, data analysts), and how | Methods: Blinding and assignment of groups | |
|  | | | 17b | If blinded, circumstances under which unblinding is permissible, and procedure for revealing a participant’s allocated intervention during the trial | Methods: Blinding and assignment of groups | |
| **Methods: Data collection, management, and analysis** | | | | |  | |
| Data collection methods | | | 18a | Plans for assessment and collection of outcome, baseline, and other trial data, including any related processes to promote data quality (eg, duplicate measurements, training of assessors) and a description of study instruments (eg, questionnaires, laboratory tests) along with their reliability and validity, if known. Reference to where data collection forms can be found, if not in the protocol | Data collection and analysis: Data collection and Data management | |
|  | | | 18b | Plans to promote participant retention and complete follow-up, including list of any outcome data to be collected for participants who discontinue or deviate from intervention protocols | Data collection and analysis: Data collection | |
| Data management | | | 19 | Plans for data entry, coding, security, and storage, including any related processes to promote data quality (eg, double data entry; range checks for data values). Reference to where details of data management procedures can be found, if not in the protocol | Data collection and analysis: Data management | |
| Statistical methods | | | 20a | Statistical methods for analysing primary and secondary outcomes. Reference to where other details of the statistical analysis plan can be found, if not in the protocol | Methods:  Statistical analysis | |
|  | | | 20b | Methods for any additional analyses (eg, subgroup and adjusted analyses) | Methods:  Statistical analysis | |
|  | | | 20c | Definition of analysis population relating to protocol non-adherence (eg, as randomised analysis), and any statistical methods to handle missing data (eg, multiple imputation) | Methods:  Statistical analysis | |
| **Methods: Monitoring** | | | | |  | |
| Data monitoring | | | 21a | Composition of data monitoring committee (DMC); summary of its role and reporting structure; statement of whether it is independent from the sponsor and competing interests; and reference to where further details about its charter can be found, if not in the protocol. Alternatively, an explanation of why a DMC is not needed | Methods: Method monitoring | |
|  | | | 21b | Description of any interim analyses and stopping guidelines, including who will have access to these interim results and make the final decision to terminate the trial | - | |
| Harms | | | 22 | Plans for collecting, assessing, reporting, and managing solicited and spontaneously reported adverse events and other unintended effects of trial interventions or trial conduct | - | |
| Auditing | | | 23 | Frequency and procedures for auditing trial conduct, if any, and whether the process will be independent from investigators and the sponsor | - | |
| Ethics and dissemination | | | | |  | |
| Research ethics approval | | | 24 | Plans for seeking research ethics committee/institutional review board (REC/IRB) approval | Methods: Ethical considerations | |
| Protocol amendments | | | 25 | Plans for communicating important protocol modifications (eg, changes to eligibility criteria, outcomes, analyses) to relevant parties (eg, investigators, REC/IRBs, trial participants, trial registries, journals, regulators) | ~~-~~ | |
| Consent or assent | | | 26a | Who will obtain informed consent or assent from potential trial participants or authorised surrogates, and how (see Item 32) | Methods:  Participant timeline | |
|  | | | 26b | Additional consent provisions for collection and use of participant data and biological specimens in ancillary studies, if applicable | Data collection and analysis: Data management | |
| Confidentiality | | | 27 | How personal information about potential and enrolled participants will be collected, shared, and maintained in order to protect confidentiality before, during, and after the trial | Data collection and analysis: Data management | |
| Declaration of interests | | | 28 | Financial and other competing interests for principal investigators for the overall trial and each study site | Conflict of interest | |
| Access to data | | | 29 | Statement of who will have access to the final trial dataset, and disclosure of contractual agreements that limit such access for investigators | Methods:  Ethical considerations | |
| Ancillary and post-trial care | | | 30 | Provisions, if any, for ancillary and post-trial care, and for compensation to those who suffer harm from trial participation | - | |
| Dissemination policy | | | 31a | Plans for investigators and sponsor to communicate trial results to participants, healthcare professionals, the public, and other relevant groups (eg, via publication, reporting in results databases, or other data sharing arrangements), including any publication restrictions | Methods:  Dissemination | |
|  | | | 31b | Authorship eligibility guidelines and any intended use of professional writers | - | |
|  | | | 31c | Plans, if any, for granting public access to the full protocol, participant-level dataset, and statistical code | - | |
| Appendices | | |  |  |  | |
| Informed consent materials | | | 32 | Model consent form and other related documentation given to participants and authorised surrogates | - | |
| Biological specimens | | | 33 | Plans for collection, laboratory evaluation, and storage of biological specimens for genetic or molecular analysis in the current trial and for future use in ancillary studies, if applicable | Methods: Ethical considerations  Data management | |
| **Data category** | **Information**[**^32^**](https://www.spirit-statement.org/spirit-statement/references#32) | | | |  |  |
| Primary registry and trial identifying number | Clinical Trials Registry (CTR)  Trial identifying number: NCT06277583 (https://clinicaltrials.gov/study/NCT06277583) | | | |  |  |
| Date of registration in primary registry | 18 February 2024 | | | |  |  |
| Secondary identifying numbers | National University of Singapore ethics committee (NUS-IRB-2023-191) | | | |  |  |
| Source(s) of monetary or material support | Singapore Ministry of Health’s National Medical Research Council | | | |  |  |
| Primary sponsor | National University of Singapore | | | |  |  |
| Secondary sponsor(s) | Not applicable | | | |  |  |
| Contact for public queries | ZS, MSc [shizhye@nus.edu.sg]  CC, PhD [[cynchen@nus.edu.sg](mailto:cynchen@nus.edu.sg)] [(65) 6601 5526]  https://classic.clinicaltrials.gov/ct2/show/NCT06277583 | | | |  |  |
| Contact for scientific queries | ZS, MSc [shizhye@nus.edu.sg]  CC, PhD [[cynchen@nus.edu.sg](mailto:cynchen@nus.edu.sg)] [(65) 6601 5526] | | | |  |  |
| Public title | Urban Care Farming on Living Well and Productive Engagement of Older Adults | | | |  |  |
| Scientific title | Effects of Urban Care Farming on Promoting Wellbeing and Productive Engagement for Older Adults: A Randomised Controlled Trial | | | |  |  |
| Countries of recruitment | Singapore | | | |  |  |
| Health condition(s) or problem(s) studied | Aging and behaviors on biopsychosocial health | | | |  |  |
| Intervention(s) | Behavioral intervention, complex intervention, urban care farming | | | |  |  |
| Key inclusion and exclusion criteria | Ages eligible for study: 50-85 years Sexes eligible for study: All Accepts healthy volunteers: yes Inclusion criteria: (1) aged 50 to 85 years; (2) willing to take part in either the intervention or waitlist control arm; (3) agree for their data to be used for research purposes; (4) ambulant and able to carry out some tasks independently.  Exclusion criteria: (1) not willing to participate in the data collection (participants can opt not to answer some questions in the questionnaire but have to consent to the other data collection methods, e.g., Blood taking/test); (2) Refusal to give informed consent; (3) are unable to give consent independently; (4) have a disability of the upper or lower body that limits their mobility; (5) have medical conditions which in the opinion of the research/investigative team, would compromise (or interfere with) their ability to participate in the study, (6) had a recent severe medical episode. | | | |  |  |
| Study type | Interventional Allocation: randomized Intervention model: parallel Masking: Assessors were blinded to participant group allocation at baseline, and 6-month and 12-month follow-up, where feasible.  Primary purpose: Prevention | | | |  |  |
| Date of first enrolment | 15/03/2024 | | | |  |  |
| Target sample size | 138 | | | |  |  |
| Recruitment status | Completed | | | |  |  |
| Primary outcome(s) | Examine the effects of urban care farming interventions, i.e., farming, horticultural, and education program, on older adults’ quality of life (i.e., WHOQOL-BREF). | | | |  |  |
| Key secondary outcomes | Examine the effects of urban care farming interventions, i.e., farming, horticultural, and education program, on older adults physical health (e.g., blood pressure, frailty, , lipids biomarkers, inflammatory biomarkers), psychosocial health (e.g., social network, loneliness, sense of community, resilience and coping, psychological stress), dietary (e.g., attitude and behavior towards consumption of fruits and vegetables), and cognition (e.g., Functional near-infrared spectroscopy and MoCA). | | | |  |  |
